# Postoperative Pulmonary Function and Structural Remodelling After Lobectomy in Patients With and Without Chronic Obstructive Pulmonary Disease

**DOI:** 10.1093/icvts/ivag068

**Published:** 2026-03-02

**Authors:** Sanae Kuroda, Tappei Shomoto, Yuki Nishioka, Nahoko Shimizu, Keiichi Morita, Wataru Nishio

**Affiliations:** Division of Chest Surgery, Hyogo Cancer Center, Akashi 673-8558, Japan; Division of Chest Surgery, Hyogo Cancer Center, Akashi 673-8558, Japan; Division of Chest Surgery, Hyogo Cancer Center, Akashi 673-8558, Japan; Division of Chest Surgery, Hyogo Cancer Center, Akashi 673-8558, Japan; Division of Chest Surgery, Hyogo Cancer Center, Akashi 673-8558, Japan; Division of Chest Surgery, Hyogo Cancer Center, Akashi 673-8558, Japan

**Keywords:** cluster analysis, lobectomy, pulmonary function, chronic obstructive pulmonary disease, 3-dimensional computed tomography

## Abstract

**Objectives:**

Quantitative assessment of lung structure provides insights beyond conventional postoperative function prediction. This study examined how preoperative chronic obstructive pulmonary disease (COPD) status and emphysema distribution influence postoperative pulmonary function and structural remodelling using 3-dimensional computed tomography (3D-CT) cluster analysis.

**Methods:**

We retrospectively analysed 426 lobectomy cases performed between 2018 and 2023. Patients were stratified into the COPD and non-COPD groups. Predicted postoperative FEV1.0 was estimated using 3D-CT volumetry, and the measured-to-predicted FEV1.0 ratio (MPFR) was calculated. Structural parameters, including *D*-value (reflecting alveolar complexity) and low-attenuation area (LAA), were measured pre- and postoperatively using 3D-CT. MPFR, % change in *D*-value (%*D*-value), and LAA (%LAA) were compared between the groups using Mann-Whitney *U* tests. Subgroup analysis was performed based on whether the resected lobe had a higher or lower *D*-value than the whole lung.

**Results:**

Patients with COPD exhibited a significantly higher MPFR than those without COPD (117.9% vs 110.7%, *P* < .001). %*D*-value did not differ significantly between the groups (99.7% vs 98.1%, *P* = .476), whereas %LAA was significantly higher in non-COPD patients (134.9% vs 117.6%, *P* = .005). In subgroup analyses according to the presence of emphysematous resected lobes, MPFR and %*D*-value did not differ between the groups, and no significant difference in %LAA was observed.

**Conclusions:**

Patients with COPD maintain better-than-predicted postoperative function without additional structural loss, whereas non-COPD patients show volume-driven increases in LAA. Integrating functional and structural 3D-CT indexes—MPFR, *D*-value, and LAA—enables a comprehensive evaluation of postoperative lung remodelling, potentially improving risk stratification and surgical planning.

**Clinical registration number:**

G-441; approved on May 12, 2025.

## Graphical abstract

**Figure:**
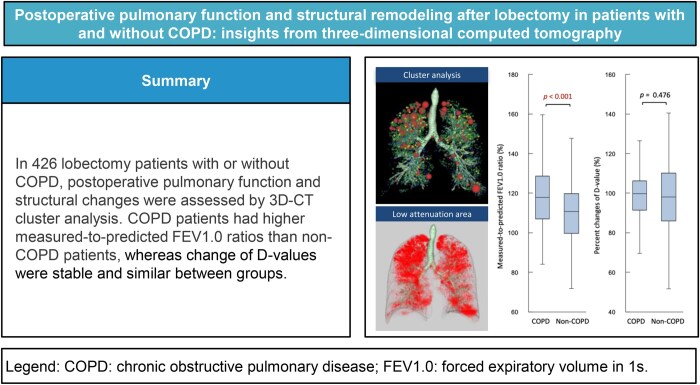

## INTRODUCTION

Chronic obstructive pulmonary disease (COPD) is a common comorbidity in patients undergoing lung resection and is associated with an increased perioperative risk and decreased pulmonary function.^1–3^ An accurate prediction of postoperative function is important for surgical planning and patient selection, particularly in lobectomy cases.^4^ Conventionally, postoperative pulmonary function has been assessed using anatomical segment counting or perfusion scintigraphy.^5–7^ However, these methods do not fully reflect regional structural differences such as emphysema severity.

Three-dimensional computed tomography (3D-CT) scan has enabled a detailed quantitative analysis of lung structure, including parameters such as low-attenuation area (LAA) and *D*-value.^8–11^ LAA reflects the extent of emphysema. Meanwhile, *D*-value, derived via low-attenuation cluster analysis, characterizes the size distribution of emphysematous regions based on a power law. When plotted on a log-log scale, LAA cluster sizes approximate a straight line, and the slope of this line—referred to as the *D*-value—becomes flatter with emphysema progression. This reflects increased heterogeneity and architectural destruction in the lung parenchyma. Thus, the *D*-value can be an objective, comprehensive indicator of alveolar complexity and emphysematous tissue damage.^12^

A volume reduction effect has been suggested after resection of emphysematous lung tissues.^13–16^ However, its impact on global lung structure and function has not been comprehensively evaluated using such quantitative imaging tools. Further, the association between preoperative functional status and postoperative structural remodelling remains unclear.

This study investigated postoperative functional and structural changes in patients with and without COPD undergoing lobectomy. Further, it evaluated the association between preoperative forced expiratory volume in 1 s (FEV1.0) and the discrepancy between actual and predicted postoperative pulmonary function, and changes in *D*-value and LAA using 3D-CT cluster analysis to validate how functional outcomes and structural compensation differ based on the underlying disease and characteristics of the resected lobe.

## METHODS

### Ethics statement

The Institutional Review Board of Hyogo Cancer Center approved this study on May 12, 2025 (approval number: G-441).

This study involved the analysis of stored clinical and imaging data obtained during routine clinical care.

The collection and use of these data were approved by the institutional review board and conducted in accordance with the Declaration of Helsinki and the World Medical Association Declaration of Taipei.

The requirement for individual informed consent was waived because of the retrospective design of the study.

### Selection of patients

Consecutive patients who underwent lobectomy at the Hyogo Cancer Center from April 2018 to December 2023 were investigated. Finally, 723 cases were included after excluding sleeve lobectomies, bilobectomies, and cases with neighbouring organ complications. Of them, 426 underwent lung function tests and 3D-CT scan before and 6 months after surgery (**Figure S1**). Postoperative spirometry and 3D-CT were not uniformly performed in all patients at the 6-month follow-up. Only those who underwent both of these examinations at 6 months were included in our analysis. The participants were divided into the COPD and non-COPD groups. COPD was defined as a FEV1.0/forced vital capacity ratio of <0.7. In patients with suspected obstructive impairment, COPD classification in this study was based on post-bronchodilator pulmonary function test results after the initiation of inhaled therapy.

Data on the demographic characteristics of the patients, pulmonary function, surgical factors, diagnosis, and postoperative complications (evaluated using the Clavien-Dindo classification) were collected and analysed. Patients with missing data were excluded from the analysis.

### CT scan and 3D lung image reconstruction

Chest CT scan was performed using 16- or 80-slice multidetector scanners (Toshiba Medical Systems, Otawara, Japan) during a single breath-hold at full inspiration. The settings were as follows: 130 kVp/150 mAs with 1-mm slices (16-slice) and 120 kVp/390-500 mAs with 0.5-mm slices (80-slice).

Three-dimensional imaging was reconstructed from the CT scan data using the SYNAPSE VINCENT 3D-CT rendering software (Fujifilm Corporation, Tokyo, Japan). In addition to the capability of reconstructing 3D images of the pulmonary vasculature, bronchial trees, and volumes, this software program enables the 3D calculation of % lung volumes <−950 HU (LAA%). In addition, cluster size and the number of LAA in pulmonary emphysema can be automatically calculated with low-attenuation voxels tracked in CT slices.

### Overview of the analytical approach

Our approach combines the ratio of actual-to-predicted postoperative FEV1.0 with 3D-CT-derived *D*-value and LAA to capture both functional preservation and structural remodelling after lobectomy. Because these indices represent complementary aspects of lung physiology, their combined assessment provides a more intuitive and clinically meaningful understanding of postoperative compensatory changes.

### Measurement of *D*-value via cluster analysis

**Figure 1.**
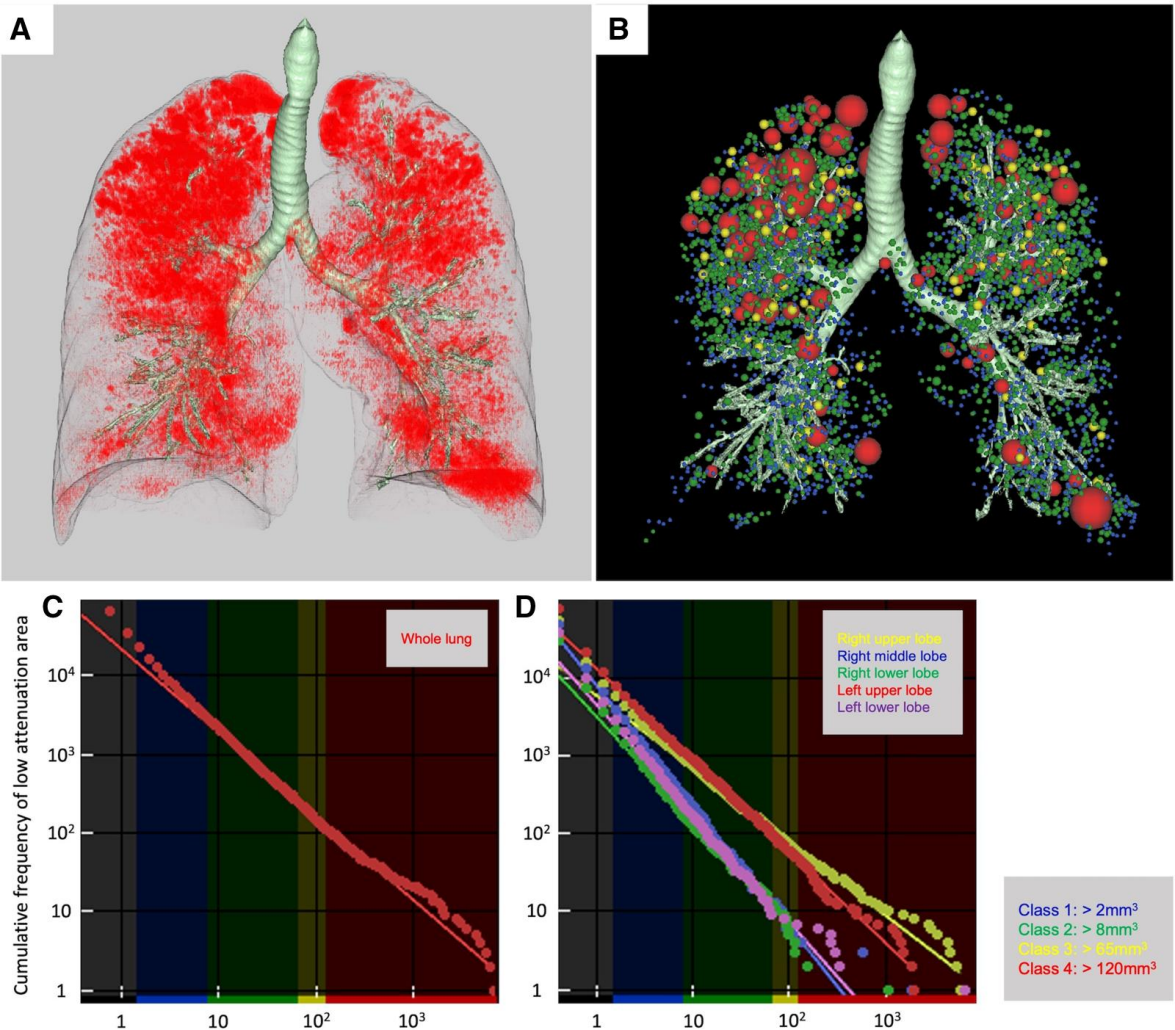
(A) LAAs identified on preoperative CT scan using the SYNAPSE VINCENT software. (B) Three-dimensional cluster analysis of LAAs, in which clusters were classified into 4 size-based categories (class 1, blue: >2 mm^3^; class 2, green: >8 mm^3^; class 3, yellow: >65 mm^3^; and class 4, red: >120 mm^3^) and colour-coded according to their size. (C, D) Log-log plots of cumulative frequency versus cluster size for the whole lung (C) and each individual lung lobe (D), with regression lines fitted to calculate the *D*-values, which corresponded to the slopes of the lines. The presented case showed preoperative imaging of a patient scheduled for right upper lobectomy. This patient belonged to the COPD group and was classified under the emphysematous resected lobe group. Abbreviations: COPD, chronic obstructive pulmonary disease; LAA, low-attenuation area.

As shown in **Figure 1**, LAA and size-based LAA clusters (**Figure 1A**) were extracted using the SYNAPSE VINCENT software. The cumulative size distribution of LAA clusters was assumed to follow a power-law distribution, characterized by an exponent known as the *D*-value, which was expressed as follows:


$$Y=KX^{-}{\;}^{D},$$

where *X* is the LAA cluster size, *Y* is the cumulative frequency, and *K* is a constant.

**Figure 1B** depicts the results of the 3D cluster analysis. The LAA clusters were classified into 4 size-based categories (class 1: >2 mm^3^, class 2: >8 mm^3^, class 3: >65 mm^3^, and class 4: >120 mm^3^) and visualized in different colours according to their size.

The *D*-value was derived using linear regression, calculated as the slope of the straight line on a log-log plot. In **Figure 1C**, the regression line was fitted using all clusters in the whole lung. Meanwhile, in **Figure 1D**, separate regression lines were fitted for each lobe. A higher *D*-value reflected preservation of the complexity of alveolar architecture, whereas a lower *D*-value indicated the predominance of large, low-attenuation clusters, which are indicative of progressive alveolar destruction.^11^

### Data analysis

FEV1.0 was measured using spirometry before and 6 months after surgery. Whole lung and lobe volumes were assessed using 3D-CT scan at the same time points.

The predicted postoperative FEV1.0 was calculated using the following formula:


preoperative FEV1.0×(whole lung volume-resection lobe volume)/whole lung volume (mL).

This CT-based volumetric approach has been reported to provide comparable or superior accuracy to conventional segment counting methods.^5^^,^^7^ Accordingly, this method was selected to ensure consistency with the structural indices evaluated in the present study.

The measured-to-predicted FEV1.0 ratio (MPFR) was calculated using the following formula:


actual postoperative FEV1.0/predicted postoperative FEV1.0 × 100 (%).

Because MPFR is calculated as a ratio of the actual postoperative FEV1.0, as measured by spirometry, to the predicted postoperative FEV1.0, estimated using 3D-CT volumetry, it reflects functional preservation rather than functioning as an independent parameter of pulmonary function.

*D*-values and LAA% were obtained from preoperative and 6-month postoperative 3D-CT scan images for the whole lung and each lobe. The rates of change in *D*-value and LAA% (%*D*-value and %LAA) were calculated using the following formula:


postoperative value/preoperative value × 100 (%).

The MPFR, %*D*-value, and %LAA of the COPD and non-COPD groups were compared.

Further, patients were classified into 2 subgroups based on the difference between whole lung and resected lobe *D*-values (the whole lung *D*-value minus the resected lobe *D*-value). Patients with a positive difference were assigned to the emphysematous resected lobe group, indicating more severe emphysema in the resected lobe than in the whole lung. Patients with a negative difference were assigned to the non-emphysematous resected lobe group. The MPFR, %*D*-value, and %LAA of these subgroups were compared to assess the impact of localized emphysema severity on postoperative pulmonary function and structural changes.

### Statistical analysis

All statistical analyses were performed with EZR 1.51 (Saitama Medical Center, Jichi Medical University, Saitama, Japan), which is a graphical user interface for R (R Foundation for Statistical Computing).^17^ The overall significance level was set at *P *< .05.

The patient variables are presented as frequencies and percentages or means ± SDs for continuous data. We made 2-group comparisons using unpaired *t*-tests for continuous data and *χ*^2^ tests for categorical data.

Mann-Whitney *U* tests were used to compare the MPFR, %*D*-value, and %LAA between the COPD and non-COPD groups, as well as between the emphysematous and non-emphysematous resected lobe groups. As these 3 outcomes were evaluated simultaneously as related measures, Bonferroni correction was applied to account for multiple testing. The threshold for statistical significance was adjusted to *P* < .017 (.05/3) for these comparisons. Other comparisons were treated as single, independent tests, and the conventional threshold of *P* < .05 was used.

We performed a supplementary multivariate linear regression for MPFR, adjusted for age, sex, smoking history, and preoperative FEV1.0. The adjusted results are presented in **Table S1**.

## RESULTS

### Surgical outcomes

In total, 426 patients were enrolled in this study. **Table 1** shows the characteristics of the patients. The COPD and non-COPD groups did not significantly differ in terms of surgical approach (thoracoscopic vs open thoracotomy) or the location of the resected lobe. However, patients with COPD were older, more likely to be male, and had a higher cumulative smoking exposure. The COPD group had significantly lower pulmonary function parameters than the non-COPD group.

**Table 1.** Patient Characteristics

Variables	COPD group	Non-COPD group	*P*-value
	*n* = 129	*n* = 297	
Age, years	74.0 ± 6.5	71.2 ± 9.2	.002
Sex			<.001
Male/female	101 (78.3)/28 (21.7)	166 (55.9)/131 (44.1)	
Smoking history (PY)	45.8 ± 32.3	20.8 ± 23.3	<.001
Preoperative VC, mL	3346 ± 752	3101 ± 794	.003
Preoperative FEV1.0, mL	1897 ± 488	2046 ± 565	<.001
Preoperative FEV1.0/FVC, %	61.8 ± 6.6	77.1 ± 5.5	<.001
GOLD classification			
Grade 1/2/3	69 (53.5)/57 (44.2)/3 (2.3)		
Preoperative whole lung *D*-value	1.4 ± 0.3	1.7 ± 0.4	<.001
Preoperative whole lung LAA, %	13.3 ± 8.7	8.0 ± 6.2	<.001
Surgical approach			
Open/VATS or RATS	121 (93.8)/8 (6.2)	281 (94.6)/16 (5.4)	.819
Operating time, minutes	222 ± 60	207 ± 56	.020
Blood loss, mL	65 ± 109	47 ± 84	.061
Resected lobe			.952
RUL/RML/RLL	51 (39.5)/8 (6.2)/26 (20.2)	121 (40.7)/19 (6.4)/65 (21.9)	
LUL/LLL	21 (16.3)/23 (17.8)	48 (16.2)/44 (14.8)	
Clavien-Dindo classification			.201
Grade 1/2	3 (20.0)/3 (20.0)	1 (2.6)/12 (31.6)	
Grade 3 A/3B	8 (53.3)/1 (6.7)	22 (57.8)/3 (7.9)	
Histology			.261
Lung cancer	128 (99.2)	283 (95.3)	
Metastases	1 (0.8)	11 (3.7)	
Lymphoma	0 (0.0)	1 (0.3)	
Benign diseases	0 (0.0)	2 (0.7)	
Tumour size, mm	29 ± 12	27 ± 13	.147
Duration of chest drainage, days	3.9 ± 3.7	3.2 ± 2.8	.031
Post/preoperative whole lung volume, %	90.7 ± 11.0	90.8 ± 12.5	.887

Values are *n*, mean ± SD, or *n (*%).

Abbreviations: FEV1.0, forced expiratory volume in 1 s; FVC, forced vital capacity; LAA, low-attenuation area; LLL, left lower lobectomy; LUL, left upper lobectomy; PY, pack year; RATS, robot-assisted thoracoscopic surgery; RLL, right lower lobectomy; RML, right middle lobectomy; RUL, right upper lobectomy; VATS, video-assisted thoracic surgery; VC, vital capacity.

The COPD group had a longer surgical duration (215 vs 197 min, *P* = .020) and chest drainage duration than the non-COPD group (3.9 vs 3.2 days, *P* = .031). There was no significant difference in the incidence of postoperative complications based on the Clavien-Dindo classification. Preoperative structural analysis using CT scan revealed that the COPD group had a significantly lower whole lung *D*-value (1.36 vs 1.57, *P* < .001) and a significantly higher LAA (11.1 vs 6.4%, *P* < .001) than the non-COPD group.

### Comparison of functional and structural parameters between the COPD and non-COPD groups

**Figure 2.**
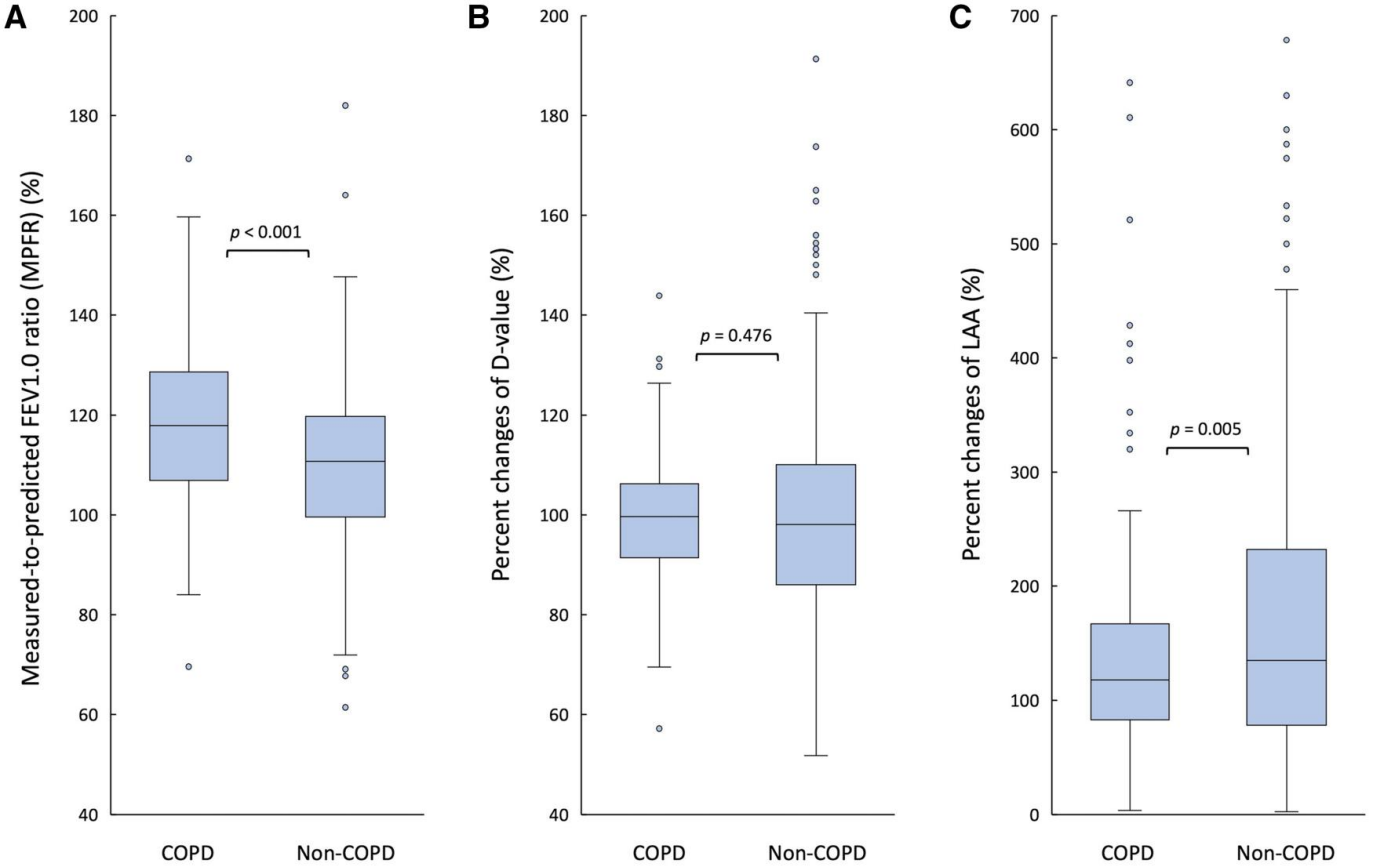
Comparison of Functional and Structural Parameters Between the COPD and Non-COPD Groups. (A) The measured-to-predicted FEV1.0 ratio (MPFR). (B) The rates of percent change in *D*-value (%*D*-value). (C) The rates of percent change in low-attenuation area (%LAA). Statistical significance was evaluated with Bonferroni correction (adjusted *P* < .017). Abbreviation: COPD, chronic obstructive pulmonary disease.

Patients with COPD had a significantly higher MPFR than those without (117.9% vs 110.7%, *P *< .001) (**Figure 2A**). There was no significant difference in terms of the %*D*-value between the 2 groups (99.7% vs 98.1%, *P *= .476) (**Figure 2B**). In contrast, the non-COPD group had a significantly higher %LAA than the COPD group (134.9% vs 117.6%, *P *= .005) (**Figure 2C**). These differences in the MPFR and %LAA remained statistically significant after Bonferroni correction (adjusted significance threshold *P *< .017). In our supplementary multivariate analysis, COPD remained independently associated with higher MPFR after adjustment for potential confounders (**Table S1**).

### Functional and structural parameters based on the emphysematous status of the resected lobes

**Figure 3.**
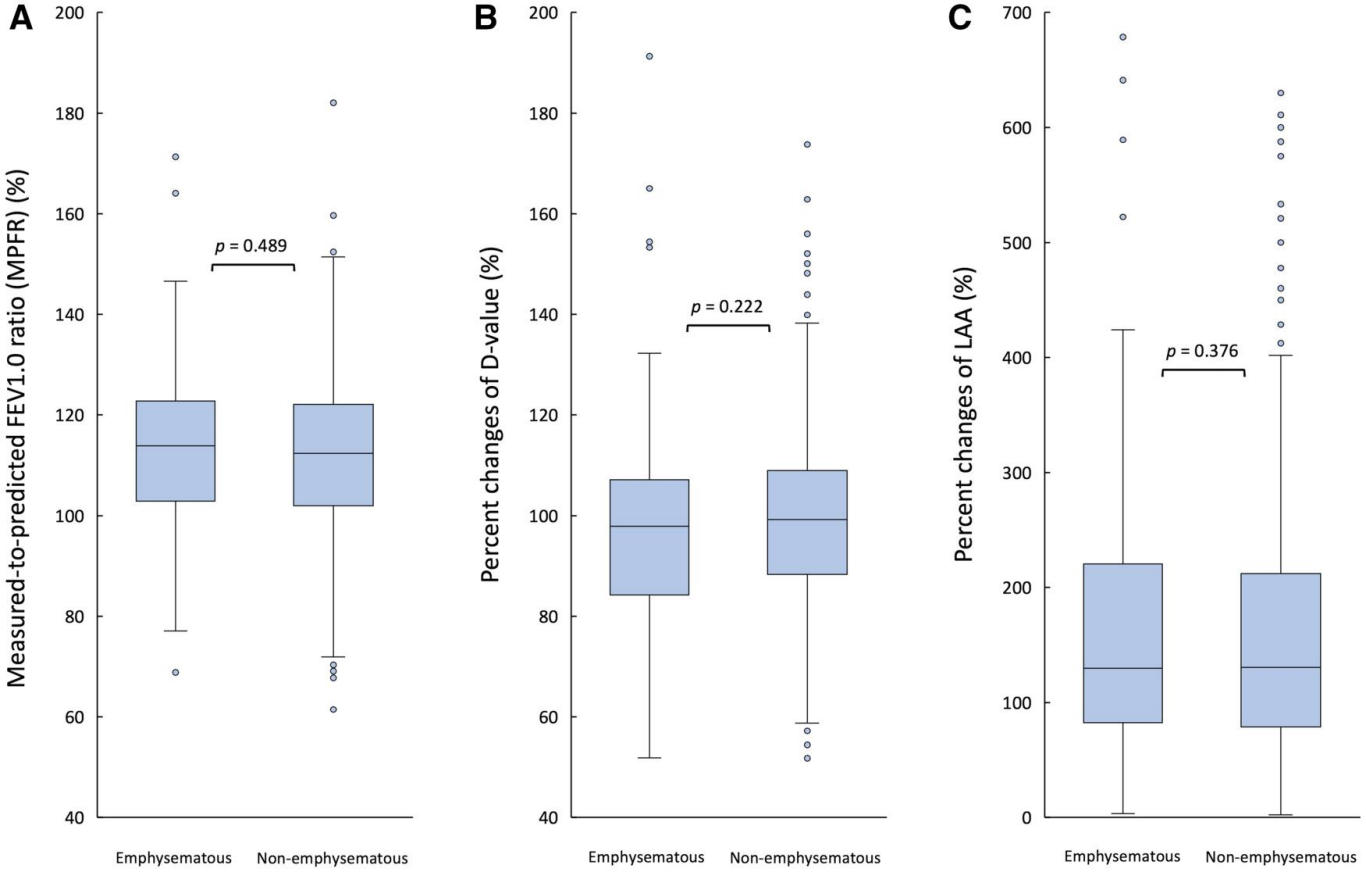
Comparison of Functional and Structural Parameters Between the Emphysematous and Non-Emphysematous Resected Lobe Groups. (A) The measured-to-predicted FEV1.0 ratio (MPFR). (B) The rates of percent change in *D*-value (%*D*-value). (C) The rates of percent change in low-attenuation area (%LAA). Statistical significance was evaluated with Bonferroni correction (adjusted *P* < .017).

**Table S2** shows the characteristics of the emphysematous and non-emphysematous resected lobe groups. The MPFR (113.9% vs 112.3%, *P *= .489) (**Figure 3A**) and %*D*-value (97.8% vs 99.3%, *P *= .222) (**Figure 3B**) did not differ between the 2 groups. In contrast, %LAA was significantly higher in the emphysematous resected lobe group than in the non-emphysematous group (129.8% vs 130.3%, *P *= .376) (**Figure 3C**), and this difference remained significant after Bonferroni correction.

## DISCUSSION

This study showed that patients with COPD exhibited a more evident postoperative functional preservation, which is consistent with the concept of a volume reduction effect.^18–20^ However, structural analyses using *D*-value and LAA revealed distinct characteristics in postoperative remodelling, indicating different underlying mechanisms of compensation between lungs with COPD and those without.

One of the key findings was that the *D*-value, which reflects alveolar complexity and the extent of microstructural destruction, did not change significantly after surgery in either group. The *D*-value is derived from the size distribution pattern of LAA clusters, representing the heterogeneity of emphysematous changes. In patients with COPD, the lung parenchyma has already undergone substantial structural destruction, leading to a stabilized state with a lower *D*-value. Therefore, even after anatomical resection, the global architectural complexity of the remaining lung does not change significantly. Conversely, in patients without COPD, the baseline lung structure is relatively intact. Thus, anatomical resections have minimal influence on *D*-value. As a result, postoperative changes in *D*-value were not significant in both the groups, explaining the absence of significant differences.

In contrast, LAA is a more dynamic parameter that reflects volumetric and functional changes in response to surgical interventions. Interestingly, the %LAA increased significantly in the non-COPD group than in the COPD group. This likely reflects the differential compensatory mechanisms after lobectomy. In patients without COPD, the elastic recoil and expansion capacity of the residual lung tissue are preserved, leading to compensatory overexpansion and a relative increase in LAA postoperatively. By contrast, lungs with COPD are already hyperinflated with a limited capacity for further volumetric expansion. In addition, resection of severely emphysematous, non-functional lung segments may result in an effect akin to surgical lung volume reduction, leading to improved ventilation efficiency without additional structural damage.

Based on these findings, the *D*-value primarily captures qualitative structural integrity. Meanwhile, LAA is more sensitive to quantitative volumetric changes and functional shifts post-surgery. By performing evaluations using both indices, a more comprehensive assessment of the lung’s structural and functional remodelling can be achieved. In this context, the postoperative changes observed in both the *D*-value and %LAA suggest adaptive lung remodelling after lobectomy. Notably, in patients with COPD, postoperative pulmonary function was often better preserved than predicted, a finding that may be partly explained by these structural adaptations. Integrating these complementary indices with functional preservation assessed by MPFR provides a more comprehensive physiological framework for interpreting postoperative pulmonary function.

Further, even in cases where emphysema is localized, the postoperative functional retention rate (measured-to-predicted FEV1.0 ratio) remained consistent, regardless of whether the resected lobe was more emphysematous or not. This finding indicated that the compensatory capacity of the residual lung is influenced more by the overall lung condition rather than the emphysema distribution pattern within specific lobes. Therefore, postoperative function is maintained whether emphysematous or normal lung segments are resected during surgery. This result emphasizes the importance of global lung mechanics over local anatomical factors.

These findings should be interpreted in the context of the retrospective study design and potential selection bias. Accordingly, the present results are descriptive in nature and intended to provide a physiological framework for understanding postoperative lung remodelling rather than to establish causality.

This study has several limitations. First, it was a retrospective single-centre study; hence, selection bias might have been introduced. Postoperative spirometry and 3D-CT were performed selectively rather than systematically at the 6-month follow-ups. Patients who did not undergo these examinations were excluded from our analysis. This may have introduced selection bias. Second, although 3D-CT cluster analysis provides detailed structural information, it cannot completely assess functional heterogeneity such as regional ventilation and perfusion. Third, the diffusion capacity of the lung for carbon monoxide, an important indicator of gas exchange efficiency and microvascular involvement, was not evaluated in this study. This omission limited the interpretation of functional reserve and might affect the assessment of volume reduction effects, particularly in emphysema-predominant patients. Finally, postoperative changes were assessed only after 6 months, and longer-term remodelling or functional decline remains unaddressed. The definition of COPD was also based on spirometric criteria, which may not completely capture distinct emphysema- and airway-dominant phenotypes.

## CONCLUSION

In patients with COPD, postoperative functional preservation is likely attributed to a volume reduction effect. Meanwhile, patients with non-COPD exhibit compensatory overexpansion of the residual lung. Combined structural (*D*-value) and volumetric (LAA) assessments allow a comprehensive evaluation of postoperative lung remodelling, regardless of emphysema distribution patterns.

## Supplementary Material

ivag068_Supplementary_Data

## Data Availability

No new data were generated or analysed in support of this research.
