# Supplementary material for: Postoperative Pulmonary Function and Structural Remodelling After Lobectomy in Patients With and Without Chronic Obstructive Pulmonary Disease
Source: Interdiscip Cardiovasc Thorac Surg. 2026 Mar 2;41(3):ivag068. doi: 10.1093/icvts/ivag068 (PMC12989145; doi:10.1093/icvts/ivag068)
Supplement: ivag068_Supplementary_Data [file ivag068_supplementary_data.zip › Supplemental table.docx]

**Supplemental Table**

| Supplemental Table S1.  Multiple regression analysis for the measured-to-predicted FEV1.0 ratio | | | | |
| --- | --- | --- | --- | --- |
| Variables | Coefficient (β) | Standard Error | t Statistic | *p*-value |
|  |  |  |  |  |
| Age, y | 0.0004 | 0.0009 | 0.42 | 0.673 |
| Sex male (vs. female) | -0.0350 | 0.0199 | -1.76 | 0.078 |
| Smoking history (PY) | -0.0003 | 0.0003 | -0.86 | 0.392 |
| COPD yes (vs. no) | 0.1014 | 0.0182 | 5.57 | < 0.001 |
|  |  |  |  |  |
|  |  |  |  |  |
| β: regression coefficient; PY: pack-year. | | | | |

| Supplemental Table S2.  Patient characteristics of the emphysematous and non-emphysematous resected lobe groups | | | |
| --- | --- | --- | --- |
| Variables | Emphysematous  resected lobe group | Non-emphysematous  resected lobe group | *p*-value |
|  | n = 105 | n = 321 |  |
|  |  |  |  |
| Age, y | 74 [70-78] | 73 [68-77] | 0.154 |
| Sex |  |  | 0.164 |
| Male / female | 72 (68.6) / 33 (31.4) | 195 (60.7) / 126 (39.3) |  |
| Smoking history (PY) | 40.0 [0.0-54.0] | 19.5 [0.0-45.0] | < 0.001 |
| Preoperative VC, mL | 3090 [2580-3720] | 3200 [2550-3790] | 0.502 |
| Preoperative FEV1.0, mL | 2150 [1810-2560] | 2260 [1850-2680] | 0.299 |
| Preoperative FEV1.0/FVC, % | 72.0 [66.3-76.7] | 73.4 [68.4-78.7] | 0.092 |
| Preoperative  whole lung D-value | 1.46 [1.30-1.85] | 1.51 [1.33-1.75] | 0.992 |
| Preoperative  resected lobe D-value | 1.41 [1.19-1.69] | 1.82 [1.53-2.37] | < 0.001 |
| Preoperative  whole lung LAA, % | 7.9 [3.4-14.8] | 7.6 [4.3-12.8] | 0.547 |
| Preoperative  resected lobe LAA, % | 8.6 [3.1-15.8] | 7.0 [3.4-11.6] | 0.012 |
| Operating time, min | 217 [176-266] | 198 [171-235] | 0.004 |
| Blood loss, mL | 20 [10-60] | 20 [10-60] | 0.417 |
| Resected lobe |  |  | 0.266 |
| RUL / RML / RLL | 38 (36.2)/9 (8.6)/19 (18.1) | 134 (41.7)/18 (5.6)/72 (22.4) |  |
| LUL / LLL | 23 (21.9) / 16 (15.2) | 46 (14.3) / 51 (15.9) |  |
| Duration of chest drainage, days | 2 [2-3] | 2 [2-3] | 0.501 |
|  |  |  |  |
|  |  |  |  |
| Values are n, median [IQR], or n (%).  PY: pack year  VC: vital capacity; FEV1.0: forced expiratory volume in 1 s; FVC: forced vital capacity  LAA: low attenuation area  RUL: right upper lobectomy; RML: right middle lobectomy; RLL: right lower lobectomy  LUL: left upper lobectomy; LLL: left lower lobectomy. | | | |
