# Supplementary figures and images for: Postoperative Pulmonary Function and Structural Remodelling After Lobectomy in Patients With and Without Chronic Obstructive Pulmonary Disease
Source: Interdiscip Cardiovasc Thorac Surg. 2026 Mar 2;41(3):ivag068. doi: 10.1093/icvts/ivag068 (PMC12989145; doi:10.1093/icvts/ivag068)

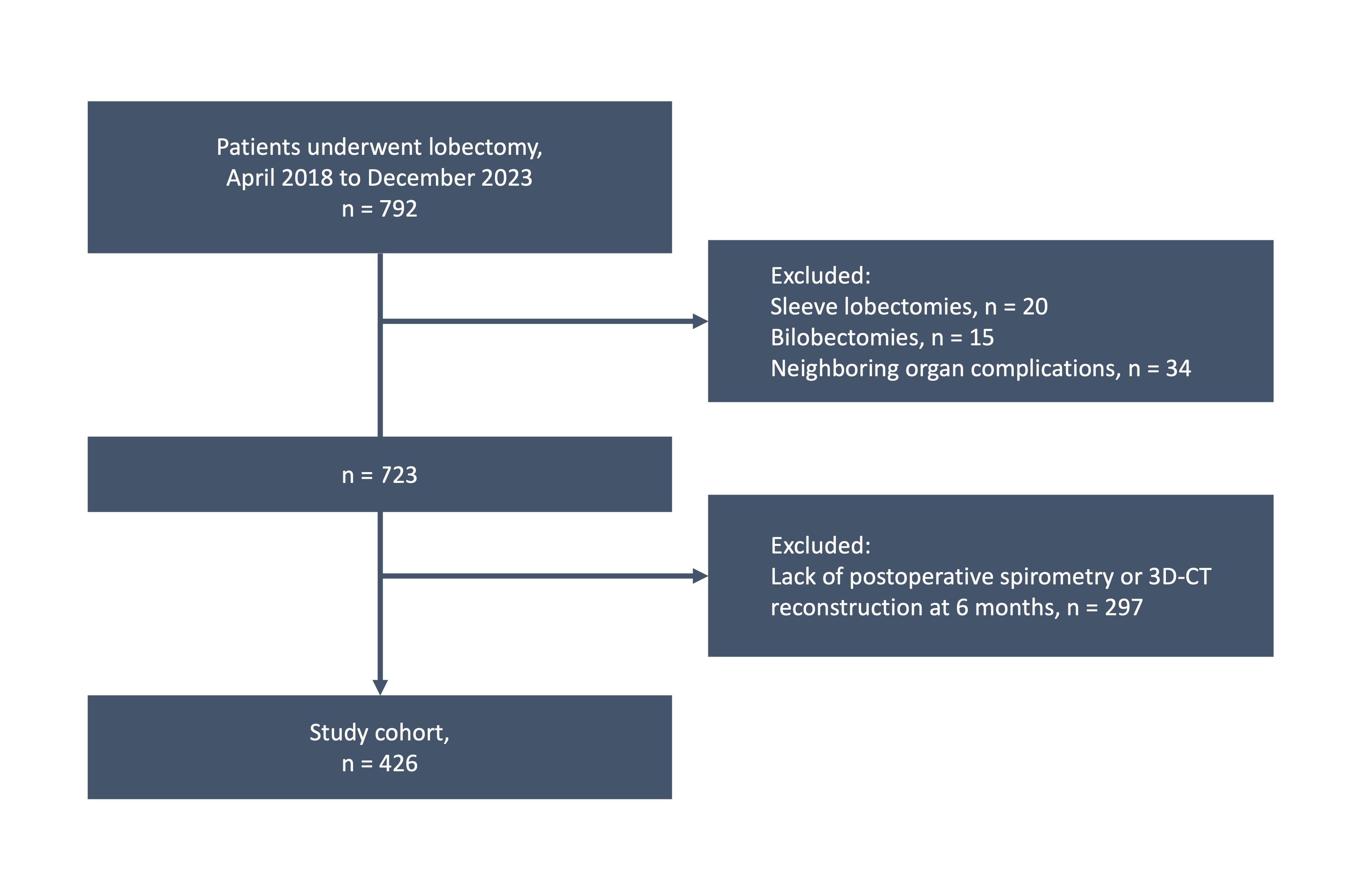

Supplement: ivag068_Supplementary_Data [file ivag068_supplementary_data.zip › Supplemental Figure1.tiff]
